# Prevention by the Natural Artocarpin of Morphological and Biochemical Alterations on UVB-Induced HaCaT Cells

**DOI:** 10.1155/2021/5067957

**Published:** 2021-07-06

**Authors:** Kunlathida Luangpraditkun, Marion Tissot, Anupong Joompang, Pensri Charoensit, François Grandmottet, Jarupa Viyoch, Céline Viennet

**Affiliations:** ^1^Department of Pharmaceutical Technology, Faculty of Pharmaceutical Sciences and Center of Excellence for Innovation in Chemistry, Naresuan University, Phitsanulok 65000, Thailand; ^2^UMR 1098 RIGHT INSERM EFS BFC, DImaCell Imaging Resource Center, University of Bourgogne Franche-Comté, Besançon 25000, France; ^3^Department of Biochemistry, Faculty of Science, Khon Kaen University, Khon Kaen 40002, Thailand; ^4^Department of Biochemistry, Faculty of Medical Science, Naresuan University, Phitsanulok 65000, Thailand

## Abstract

Natural substances have gained considerable attention for skin protection against UV light reactions. *Artocarpus altilis* plant's heartwood extract is comprised of artocarpin as a major substance, already known for its interesting biological attributes as an antimicrobial, an anti-inflammatory, an antioxidant, and a melanogenesis inhibitor. The present work clarified the mechanism of natural artocarpin (NAR) with a purity of approximately 99% against the effects of UVB-induced HaCaT keratinocyte apoptosis. The indicated results showed that NAR suppresses free radical production (ROS and nitrite) and apoptosis-related molecule activation (caspase-3, p-p53, p-p38, and NF-*κ*B p65) and secretion (TNF-*α*). Additionally, NAR prevented structural damages (nuclei condensation and fragmentation, apoptotic body formation, impaired cell adherence and round cell shape, disruption of F-actin filament, and clustering of cell death receptor CD95/Fas) and biophysical changes (plasma membrane rigidification). Thus, NAR acts directly from scavenging free radicals generated by UV and indirectly by suppressing morphological and biochemical UV-induced cell damages. Its biological effects are mainly attributed to antioxidant and antiapoptotic properties. Taken together, NAR could be considered as an effective natural product for photoprotective formulations.

## 1. Introduction

Keratinocytes (KCs) are the predominant cells in the epidermis, the outermost skin layer. An intact epidermis acts as an effective barrier that protects us from various environmental hazardous factors. Accordingly, the delicate balance between KC proliferation, differentiation, and apoptosis result in maintaining epidermal homeostasis [1–3]. KCs are important cellular targets for solar ultraviolet B (UVB) radiation that is responsible for sunburn, photoaging, immune suppression, and skin cancers [4–7]. At the cellular and molecular levels, excessive UVB exposure leads to several signaling responses involving morphological and biochemical changes on KCs [8]. Prominent events in KCs exposed to UVB are plasma membrane and DNA damages [2, 8–10]. The plasma membrane is a highly dynamic structure and is considered fluid, with rotational, translational, and transbilayer lipid movement. Its composition and organization contributes to cellular signaling and function. UV irradiation influences the membrane fluidity through the lipid environment [11]. It activates nitric oxide synthase to produce a molecule named nitric oxide (NO), and NO reduces membrane fluidity [12]. Furthermore, UVB evokes reactive oxygen species (ROS) reacting with polyunsaturated fatty acids which are essential components for the lipid membrane and inducing lipid peroxidation [13, 14]. Lipid peroxidation leads to changes in the integrity, permeability, and fluidity of the plasma membrane [15]. It has been well documented that ROS mediate the phosphorylation of protein kinases (such as p-p38) all over the sequences of mitogen-activated protein kinase (MAPK) signal transduction cascades [16]. MAPKs act as the key upstream regulators for transcription factor activities, like the nuclear factor-*κ*B (NF-*κ*B). UVB-provoked oxidative stress results in the stimulation of NF-*κ*B by p38/MAPK relating to cell death, matrix metalloprotease (MMPs) expression, and proinflammatory cytokine release including tumor necrosis factor-alpha (TNF-*α*) [17, 18].

The direct UVB photon absorption by DNA or the indirect effect by oxidative stress via free radicals and ROS brings about DNA damages (cyclobutane pyrimidine dimers (CPD) and 6-4 photoproducts (6-4 PP)). They activate, via the MAPK signaling pathway, the nuclear phosphoprotein p53 which controls cell cycle arrest and DNA repair or induces the unpaired cells to undergo apoptosis [4, 19, 20]. UVB is a proapoptotic agent through the stimulation of interrelating signaling cascades involving the activation of cysteine proteases named caspases. Caspase-3 is the most important terminal caspase essential for a series of morphological changes and execution of apoptosis [21]. Caspase activation is initiated through two major apoptotic mechanisms, the intrinsic pathway depending on the DNA damage and the activated p53 and the extrinsic pathway that occurs by the activation of the TNF family (TNF-R1, CD95 (Fas/APO-1), or TRAIL-R which are death receptors located on the cell membrane) [2, 19, 20, 22]. It has been reported that the clustering of these surface receptors can be induced by UVB, even without ligand pairing anymore [22–25].

Nowadays, many natural plant-extracted compounds are used as photoprotective agents against UV-induced skin disorders. Several reports have shown interest in flavonoids in the prevention of diseases due to their pharmacological actions, mainly their antioxidant and chelating properties [26]. Notably, it has been shown that the *Artocarpus altilis* plant possesses biological efficacy, and *in vitro* as well as *in vivo* research is focused on antioxidation [27–33], antilipid peroxidation [30, 33], anti-inflammation [30, 32], antityrosinase activity [27–29], and antiapoptotic [33] activity. Our previous data have demonstrated the photoprotection activities of the natural artocarpin (NAR) isolated from *Artocarpus altilis*'s heartwood extract in UVB-induced keratinocyte apoptosis [33]. We showed that pretreatment with NAR attenuates oxidative stress by acting as free radical scavengers—both ROS and RNS (reactive nitrogen species) were scavenged—protecting DNA, and modulating the cell cycle. In this present work, to further define the antiphotodamaging mechanism of NAR, we investigated the morphological and biochemical effectiveness of the extract on UVB-affected human HaCaT keratinocytes. We studied oxidative stress (ROS and nitric oxide (NO)), biophysical change in the plasma membrane (membrane fluidity), cell structural features (actin cytoskeleton, CD95 membrane death receptor), and expression of apoptosis-related molecules (caspase-3, TNF-*α*, p53, p38/MAPK, and NF-*κ*B). Our results showed that NAR interacts with the plasma membrane, absorbs UVB rays, and decreases UV-mediated oxidative stress, thereby modulating different signaling cascades leading to apoptosis. These findings provided significant information about the potential usefulness of NAR for UV protection against skin damages together with the prophylactic actions to restore the epidermal skin barrier function.

## 2. Materials and Methods

### 2.1. Purification of Natural Artocarpin

Natural artocarpin was purified from the heartwood of the *A. altilis* plant according to the method described in previously published papers [32, 33]. Briefly, the heartwood was chipped into small pieces and dried at 45°C until dryness. The dried heartwood was macerated with diethyl ether at room temperature for 2 cycles (2 days/cycle). The gathered extract was filtered, and the solvent was removed by using a rotary evaporator. The artocarpin was purified by using column chromatography. The silica gel (No. 60, Merck, Darmstadt, Germany) was packed into a column. Then, the column was eluted with an eluting solvent of hexane and a continuously increasing ratio of ethyl acetate. Every 5 cm^3^ of fractions was collected, and the remaining flavonoid was assessed using thin layer chromatography (TLC) (Merck, Darmstadt, Germany). Only similar TLC profiles were gathered, and the solvent was removed. The concentrated extract was stored at 4°C. All solvents were of analytical grade and purchased from Labscan Asia Co., Ltd., Thailand.

### 2.2. Determination of the Purity of the Natural Artocarpin

The content of artocarpin in the extract was determined using isocratic high-performance liquid chromatography (HPLC). The system consisted of an SPD-20A UV detector, an LC-20AP pump (Shimadzu Co., Ltd., Kyoto, Japan), and a column (with a diameter of 250 × 4.60 mm) packed with 5 *μ*m C18 (Phenomenex Gemini column). Methanol and water (80 : 20) were used as the mobile phase. Natural artocarpin was dissolved in this mobile phase. The sample was injected into column with a volume of 20 *μ*L. The isocratic mode was performed with a flow rate of 1 mL/min and monitoring at 282 nm. A calibration curve of the integrated peak area generated from standard artocarpin (BioBioPha Co., Ltd., Yunnan, China) was used to calculate the quantity of artocarpin. All mobile phases were of HPLC grade. All experiments were performed in triplicate (*n* = 3).

### 2.3. HaCaT Cell Culture

HaCaT cells were purchased from CliniSciences (France). They were cultured in Dulbecco's Modified Eagle's Medium supplemented with 10% fetal bovine serum, 10,000 U/mL penicillin, and 10 mg/mL streptomycin (all from PAN Biotech, Dominique Dutscher, France) at 37°C in a humid atmosphere containing 5% CO_2_. When cells (from passages 3 to 22) reached 70-80% confluence, they were trypsinized, planted at different densities according to the parameters studied, and incubated for 24 hr.

### 2.4. Pretreatment of HaCaT Cells with Natural Artocarpin (NAR)

NAR was isolated from fresh heartwood of the *Artocarpus altilis* plant as previously described [33]. HaCaT cells were pretreated for 24 hr with nonsupplemented medium, and NAR was then added (3.1 *μ*g/mL). NAR was dissolved in noncytotoxic DMSO concentration (<0.1% *v*/*v* as previously described).

### 2.5. UVB Exposure

Cells were exposed to UVB irradiation with an intensity of 55 mJ/cm^2^, as previously described [33]. The UV source was provided by UVB tubes (8 W, F8T5, Sankyo Denki, Japan) which radiate UV rays during 280-360 nm (peak wavelength from 305 to 315 nm). The UVB dose was determined using UV light meter (IL1700, International Light Technologies, USA). Before irradiation, the phosphate-buffered saline (PBS) was used to rinse cells for 2 times, and they were then exposed to UVB at 55 mJ/cm^2^, covered with a PBS layer. After that, cells were cultivated in nonsupplemented medium for any desired time.

### 2.6. Assessment of Oxidative Stress

#### 2.6.1. ROS Assay

A 2′,7′-dichlorofluorescein-diacetate (DCH_2_F-DA) fluorescence probe (Sigma-Aldrich, France) was employed to detect intracellular ROS levels. Briefly, HaCaT cells were cultivated in 96-well plates (1 × 10^4^ cells/well). After the cells were given NAR pretreatment for 24 hr and exposed to UVB, the cells were immediately rinsed with PBS for 2 times. Next, they were incubated with 10 *μ*M of the DCH_2_F-DA probe in nonsupplemented medium for 30 min at 37°C. Cells were rinsed with PBS for 3 times, and PBS was equally added to cover sample plates afterwards. The fluorescence intensities were measured by using a fluorimeter (Synergy H1, BioTek®, USA) with excitation as well as emission spectra of 485 and 528 nm. Images of stained cells were acquired using a fluorescence microscope (Olympus IX50, France).

#### 2.6.2. Nitrite Assay

Nitrite level was employed as a pointer of NO yield secreted into the cell culture medium by the Griess reagent (Sigma-Aldrich). Briefly, cells were cultivated in 6-well plates (1 × 10^6^ cells/well). After the cells were given NAR pretreatment for 24 hr and exposed to UVB, the cells were incubated for 24 hr. Then, an equal volume of culture supernatants (50 *μ*L) together with the Griess reagent (50 *μ*L) were blended in 96-well plates and incubated at room temperature for 15 min. The absorbance was determined by using a microplate reader at 550 nm (Multiskan FC, Thermo Fisher Scientific, France). NaNO_2_ was employed as standard for calculating the concentrations of nitrite.

### 2.7. Assessment of Apoptosis

#### 2.7.1. Caspase-3 Assays

The activity of caspase-3 was determined using the NucView® 488 Apoptosis kit (Biotium, Ozyme, France). Briefly, cells were cultivated in 96-well plates (1.0 × 10^4^ cells/well). After the cells were given NAR pretreatment for 24 hr and exposed to UVB, cells were incubated for 10 hr. Then, they were rinsed with PBS for 2 times and incubated with 10 *μ*M NucView® 488 substrate for 30 min at RT in darkness. Next, cells were rinsed with PBS for 3 times, and PBS was equally added to cover sample plates afterwards. The fluorescence signals were measured by using a fluorimeter (Synergy H1) with excitation as well as emission spectra of 485 and 515 nm. Imaging of caspase-3 activity was performed by fluorescence microscopy (Olympus IX50).

The protein quantity of active caspase-3 was investigated by an Enzyme-Linked Immunosorbent Assay (ELISA). Cells were cultivated in 6-well plates (1 × 10^6^ cells/well). After the cells were given NAR pretreatment for 24 hr and exposed to UVB, cells were incubated for 10 hr. Then, cells were rinsed with PBS, collected by scraping, and cell lysis was continued. The concentration of active caspase-3 was determined following the manufacturer's protocol (Human Active Caspase-3 Ser29 ELISA Kit, Abcam, UK). Absorbance measurements were made at 450 nm by using a microplate reader. Values were calculated on the basis of a standard curve. Active caspase-3 levels were normalized to protein contents in cell lysates (determined using the Bradford assay).

#### 2.7.2. TNF-*α* ELISA

Cells were cultivated in 6-well plates (1 × 10^6^ cells/well). After the cells were given NAR pretreatment for 24 hr and exposed to UVB, cells were incubated for 24 hr. Next, culture supernatants were collected. TNF-*α* quantification was carried out by the human TNF-*α* quantikine ELISA Kit (R*&*D Systems, France). Absorbance measurements were made at 450 nm by using a microplate reader. Values were calculated on the basis of a standard curve and were normalized to intracellular protein contents.

#### 2.7.3. p-p53, p-p38/MAPK, and NF-*κ*B p65 Expression

*(1) Immunofluorescence Microscopy Imaging*. HaCaT cells were cultivated in 8-well chamber slides (2.5 × 10^4^ cells/well). After the cells were given NAR pretreatment for 24 hr and exposed to UVB, cells were incubated for different times. Then, fixation was performed by incubating 4% paraformaldehyde solution (PFA) for 10 min at RT, and further permeabilization was done by incubating 0.1% Triton X-100 (Sigma-Aldrich) for 15 min at RT, followed by 1 hr incubation at RT in an appropriate blocking buffer. Cells were immunostained overnight at 4°C with phospho-p53 (Ser15) (16G8) Mouse mAb (p-p53) (1 : 200), phospho-p38/MAPK (Thr180/Tyr182) (28B10) Mouse mAb (p-p38) (1 : 100), and NF-*κ*B p65 (D14E12) XP® Rabbit mAb (NF-*κ*B) (1 : 75, Alexa Fluor® 488 Conjugate) (Cell Signaling Technology, Inc., USA). Then, they were incubated for 1 hr at RT with FITC or rhodamine-conjugated antibodies (1 : 75, Sigma-Aldrich) and with 4′,6-diamidine-2′-phenylindole dihydrochloride (DAPI) solution (5 *μ*L/mL, Sigma-Aldrich) for 10 min at RT. Confocal images were acquired with LSM 800 (Zeiss, France) with ×63 objective.

*(2) Flow Cytometry*. HaCaT cells were cultivated in 6-well plates (1 × 10^6^ cells/well). After the cells were given NAR pretreatment for 24 hr and exposed to UVB, cells were incubated for different times. Then, cells were gathered using trypsin and fixed with 4% PFA for 10 min at RT. Flow cytometry staining protocol used the same panel of antibodies (p-p53 diluted at 1 : 800, p-p38 diluted at 1 : 100, and NF-*κ*B p65 diluted at 1 : 50) and procedure as microcopy imaging. Isotype controls were employed in parallel to specifically endorse the occurrence of antibody matching, along with the production of the signal gates with positively tagged cells. Cell suspension was done in 2 mM EDTA/PBS before acquisition on an LSR Fortessa Flow Cytometer (Becton Dickinson, France).

### 2.8. Assessment of Structural and Biophysical Changes

#### 2.8.1. Confocal Fluorescence Microscopy

HaCaT cells were cultivated in 8-well chamber slides (2.5 × 10^4^ cells/well). After the cells were given NAR pretreatment for 24 hr and exposed to UVB, cells were incubated for different times. Then, fixation was performed by incubating 4% PFA for 10 min. For cytoskeleton as well as nucleus labeling, permeabilization was performed by incubating 0.1% Triton X-100 for 15 min at RT. They were double-stained with rhodamine-conjugated phalloidin (2.5 *μ*g/mL, Sigma-Aldrich) for 15 min and DAPI solution for 10 min at RT. For CD95/Fas membrane receptor labelling, cells were incubated for 1 hr at RT in an appropriate blocking buffer, then incubated with anti-Fas Rabbit pAb (2 *μ*g/mL, Sigma-Aldrich) overnight at 4°C, followed by incubation for 1 hr at RT with secondary anti-rabbit antibody conjugated with rhodamine (1 : 75, Sigma-Aldrich). Confocal images were acquired with LSM 800 (Zeiss) with a ×63 objective.

#### 2.8.2. Scanning Electron Microscopy

Fixed cells were dehydrated throughout the set of graded ethanol (ranging from 30 to 100%). Drying was taken by the critical point drying (CPD) method (Leica EM CPD030). Coating all samples using a thin gold layer was performed using a Quorum Q150T ES plus. Eventually, samples were scanned using a scanning electron microscope (Hitachi SU8230; Elexience, France).

#### 2.8.3. Membrane Fluidity by TMA-DPH Fluorescence Anisotropy Assay

We used the steady-state fluorescence polarization method [34]. In brief, HaCaT cells were cultivated in 6-well plates (1 × 10^6^ cells/well) and pretreated with NAR for 24 hr. Cells were gathered 2 min after UVB irradiation by gentle scraping in PBS with a cell lifter which left them undamaged. Cells were then labelled with the fluorescent probe TMA-DPH (2 *μ*M). Membrane fluidity was assessed 1 min after cell labelling on a fluorescent spectrophotometer equipped with polarizers (Perkin-Elmer, France). Samples were excited by exposure to vertically polarized light (360 nm), and emitted light was analyzed at 435 nm vertically and horizontally to the direction of the excitation. All fluorescent measurements were carried out at 23°C.

Anisotropy values (*r*) were calculated as follows:

(1) $$\begin{array}{c} r=\frac{I_{\text{VV}}-G\times I_{\text{VH}}}{I_{\text{VV}}+2G\times I_{\text{VH}}}, \end{array}$$

where *I*_VV_ is the emission intensity that is vertically measured, *I*_VH_ is the emission intensity that is horizontally measured, and *G* is the correction factor that is used for calculations in an optical system.

### 2.9. Statistics

Data are independently represented as means ± standard deviation (S.D.) of one or three of the experimentations. Each one was operated in triplicate (*n* = 3), and each group was compared using Student's unpaired *t*-test. *p* < 0.05 was regarded as significant.

## 3. Results

### 3.1. The Purity of Artocarpin in the Extract

The amount of artocarpin in the extract was 99.30 ± 0.03%*w*/*w* according to HPLC. Therefore, this extract was the purified artocarpin extract.

### 3.2. Protective Effects of NAR versus UVB-Evoked Oxidative Stress in HaCaT Cells

**Figure 1:**
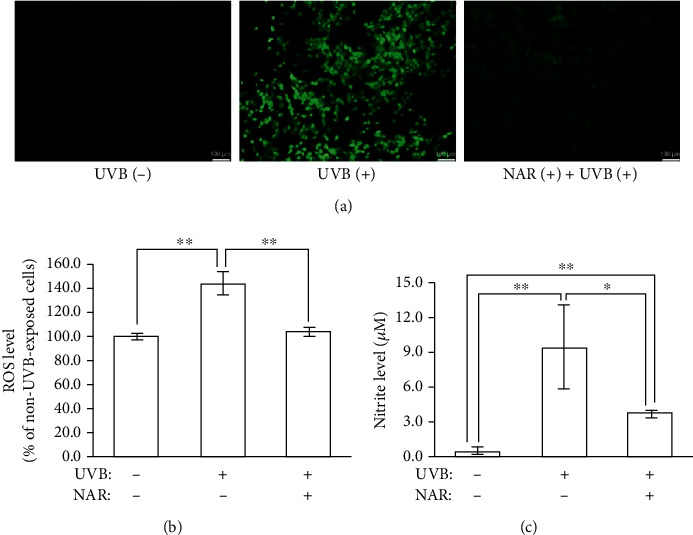
Antioxidant action by NAR to UVB-exposed HaCaT. The intracellular ROS and nitrite production were measured in HaCaT untreated or treated with NAR (3.1 *μ*g/mL) for 24 hr. Immediately after UVB irradiation (55 mJ/cm^2^), cells were incubated with the DCH_2_F-DA probe to detect ROS level by (a) fluorescence microscopy and (b) fluorimetry (excitation: 485 nm; emission: 528 nm). (c) Later on, 24 hr after UVB irradiation, media were collected, and nitrite level was measured by the Griess assay spectrophotometrically at 550 nm. Three of experimentations were operated. Each one was done in triplicate (*n* = 3). Results are mean ± S.D.^∗^*p* < 0.05 and ^∗∗^*p* < 0.01, using Student's unpaired *t*-test.

The stimulated ROS inside the cells was detected using the DCH_2_F-DA fluorescent probe. Fluorescence spectrometry data showed significantly higher ROS content in UVB-irradiated cells (143.6% ± 10.1) relative to non-UVB-irradiated cells (100% ± 2.2). A significant decrease of intracellular ROS occurred in HaCaT treated with NAR (103.9% ± 3.4) (Figure 1(b)). The results were confirmed by fluorescence microscopy observations (Figure 1(a)). NAR inhibited ROS accumulation in UVB-exposed cells.  Figure 1(c) shows the concentration of nitrite, a major oxidative metabolite of NO, assessed by a Griess reagent assay. The level of nitrite was significantly higher in UVB-exposed HaCaT cells (9.3 *μ*M ± 3.7), in contrast to non-UVB-exposed HaCaT cells (0.4 *μ*M ± 0.3) (Figure 1(c)). The NAR pretreatment significantly moderated the nitrite concentration (3.6 *μ*M ± 0.2). Overall, the results ascribed above indicated that NAR protects keratinocytes out of UVB-caused overproduction of free radicals, called oxidative stress, by scavenging ROS and NO.

### 3.3. Protective Effects of NAR versus UVB-Affected HaCaT Apoptosis

#### 3.3.1. Caspase-3 Assays

**Figure 2:**
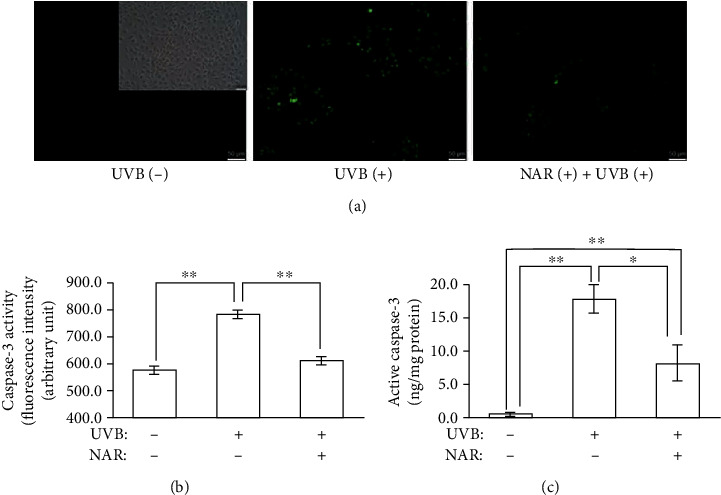
Protective effects of NAR versus UVB-affected apoptosis. The caspase-3 activity and the level of active caspase-3 protein were measured in HaCaT untreated or treated with NAR (3.1 *μ*g/mL) for 24 hr. At 10 hr after UVB irradiation (55 mJ/cm^2^), cells were gathered and the NucView® 488 substrate was added to detect caspase-3 activity by (a) fluorescence microscopy and (b) fluorimetry (excitation: 485 nm; emission: 515 nm). (c) The quantity of active caspase-3 protein was detected by human active caspase-3 Ser-29 ELISA assay. Three of experimentations were operated. Each one was done in triplicate (*n* = 3). Results are mean ± S.D. ^∗^*p* < 0.05 and ^∗∗^*p* < 0.01, using Student's unpaired *t*-test.

As caspase-3 is a crucial protease in the process of apoptosis, we investigated the effect of NAR on the activity of caspase-3 by using the NucView® 488 substrate and then fluorometric measurements. As shown in Figure 2(b), induction of apoptosis by UVB was confirmed with the increase of caspase-3 activity (787 FI ± 15 for UVB-exposed cells; 578 FI ± 12 for non-UVB-exposed cells). We observed that NAR inhibits caspase-3 activity in UVB-exposed cells (614 FI ± 19). Microscopic examination also demonstrated a marked increase of green fluorescence after UVB exposure, illustrating activation of caspase-3. Cells pretreated with NAR displayed reduced fluorescence (Figure 2(a)). We next examined active caspase-3 level by ELISA assay (Figure 2(c)). Active caspase-3 concentration was significantly increased in UVB-exposed cells (17.7 ± 2.1 ng/mg protein) compared to non-UVB-exposed cells (0.4 ± 0.2 ng/mg protein). After pretreatment with NAR, the level of active caspase-3 decreased (8.2 ± 2.8 ng/mg protein). Therefore, the protective effect of NAR against UVB-affected HaCaT apoptosis was mediated in part through caspase-3. There was a positive correlation between the level of active caspase-3 protein and its activity.

#### 3.3.2. Apoptosis-Related Molecules and Signaling Pathways

**Figure 3:**
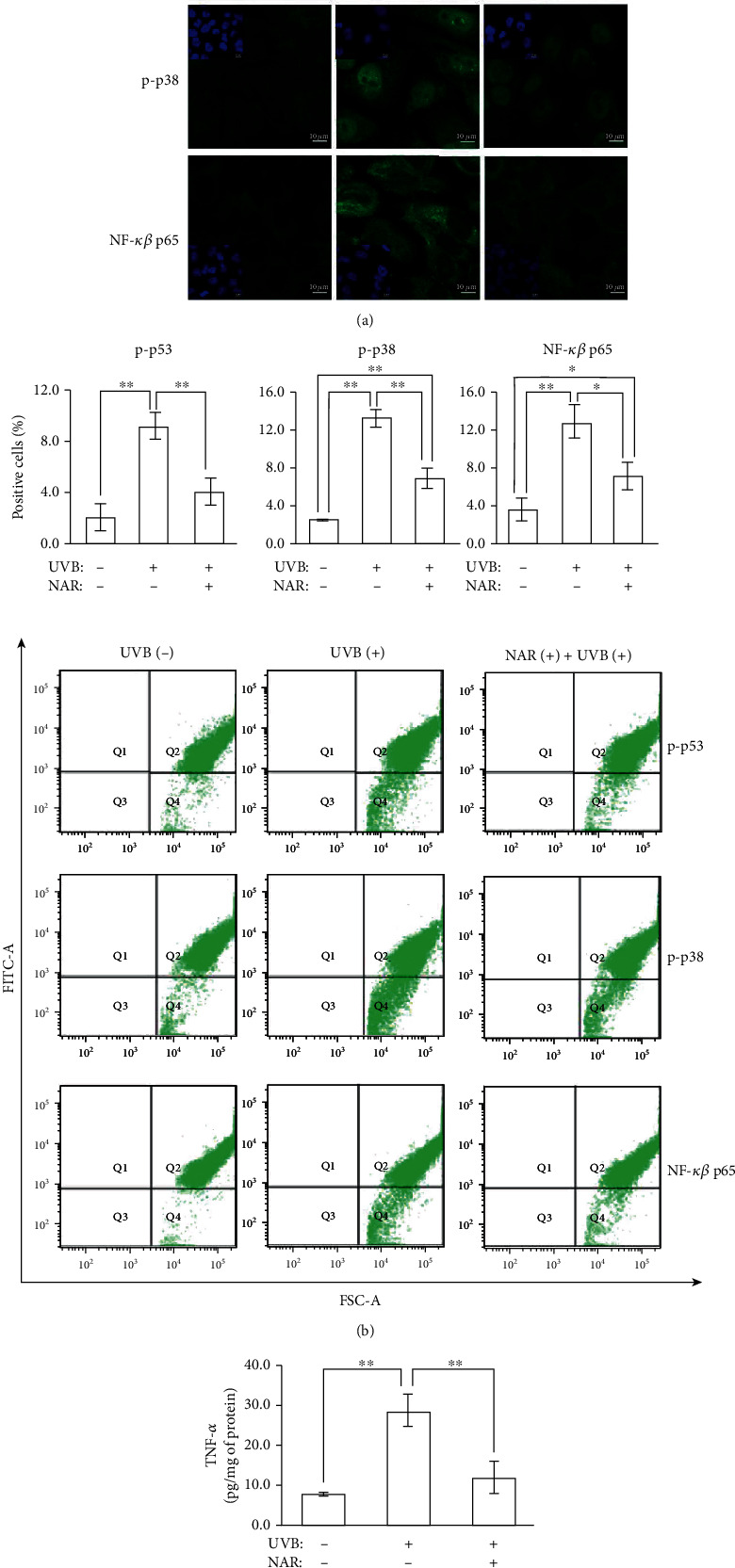
Protective effects of NAR against apoptosis-related molecules and signaling pathways. Immunofluorescence staining 6 hr after UVB irradiation (55 mJ/cm^2^) for p-p53, p-p38, and NF-*κ*B p65, in HaCaT untreated or treated with NAR (3.1 *μ*g/mL) for 24 hr. (a) Representative confocal microscopy images (63x objective magnification). (b) Flow cytometry analyses with histograms and representative p-p53, p-p38, and NF-*κ*B p65 dot plots. (c) Human TNF-*α* quantikine ELISA assay 24 hr after UVB irradiation (55 mJ/cm^2^) in supernatants of HaCaT untreated or treated with NAR. One (a, b) or three (c) experimentations were independently performed. Each one was done in triplicate (*n* = 3). Results are mean ± S.D.^∗^*p* < 0.05 and ^∗∗^*p* < 0.01, using Student's unpaired *t*-test.

We studied the expression of different apoptosis-related molecules including p-p53, p-p38, the subunit NF-*κ*B p65, and TNF-*α* (Figure 3). As some protein functions are regulated through posttranslational modifications, such as phosphorylation, we examined phosphorylated proteins. Immunofluorescence analysis by microscopy showed that p-p53, p-p38, and NF-*κ*B p65 expression was upregulated after UVB irradiation (Figure 3(a)). Similar to p-p38, the staining of p-p53 was localized in the nucleus, whereas the staining of NF-*κ*B p65 was mainly in the cytoplasm. For the NAR-pretreated UVB-exposed cells, the expression of these molecules was reduced compared to UVB-exposed cells. Flow cytometry data confirmed fluorescence microscopy imaging and therefore reported a significant effect of NAR on some particular signaling cascades transmitted and transcription factors linked to the cellular response to UVB-affected apoptosis (%p − p53 fluorescence intensity = 9.2 ± 1.0 and 4.0 ± 1.2 for UVB-irradiated vs. NAR-pretreated UVB-irradiated cells, respectively; %p − p38 fluorescence intensity = 13.2 ± 0.9 and 6.9 ± 1.2 for UVB-irradiated vs. NAR-pretreated UVB-irradiated cells, respectively; %NF − *κ*B p65 fluorescence intensity = 12.9 ± 1.8 and 7.2 ± 1.3 for UVB-irradiated vs. NAR-pretreated UVB-irradiated cells, respectively; Figure 3(b)).

Additionally, we also investigated by ELISA assay the production of TNF-*α*, a proinflammatory and apoptosis*-*inducing cytokine (Figure 3(c)). After UVB irradiation, TNF-*α* was significantly upsecreted in UVB-exposed cells (28.3 ± 4.3 pg/mg protein), compared to non-UVB-exposed cells (7.7 ± 0.3 pg/mg protein). NAR pretreatment significantly inhibited the secretion of TNF-*α* by UVB-exposed cells (11.8 ± 4.0 pg/mg protein).

### 3.4. Protective Effects of NAR versus UVB-Influenced Structural and Biophysical Changes

#### 3.4.1. Nucleus, F-Actin Cytoskeleton, and CD95/Fas Membrane Receptor

**Figure 4:**
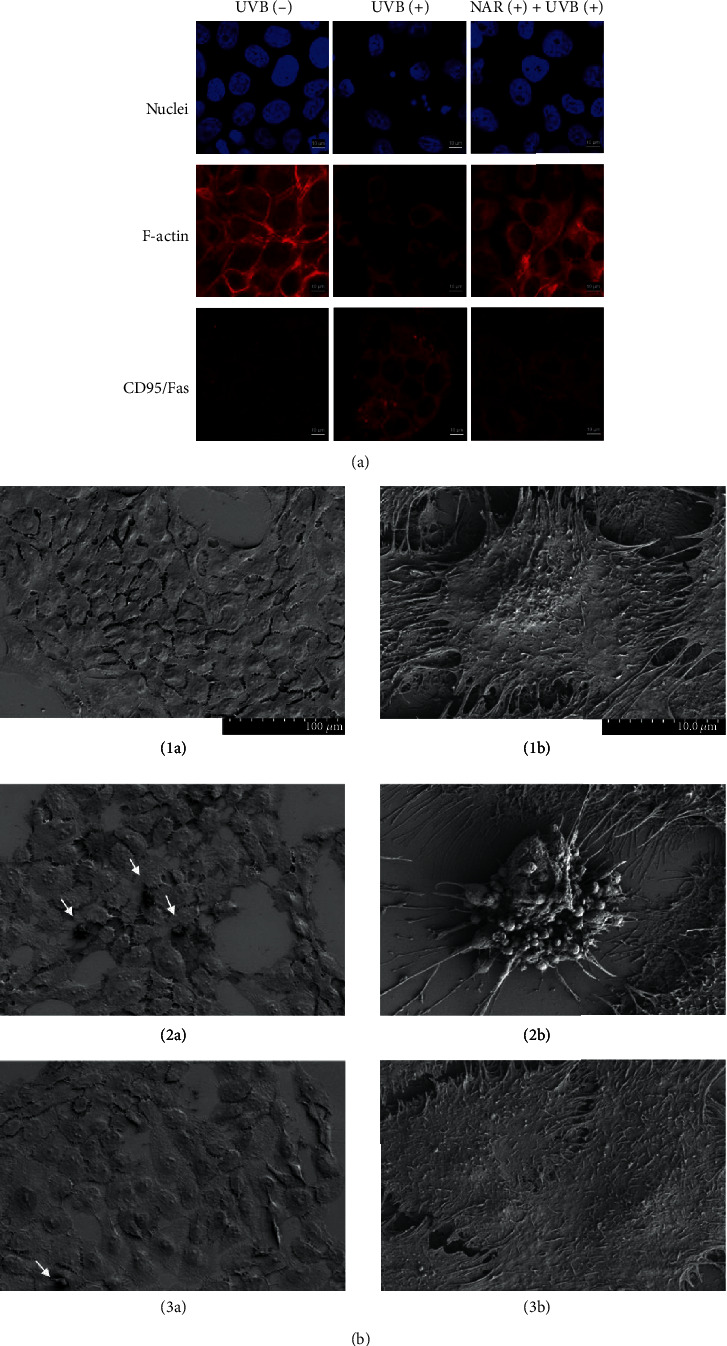
Protective effects of NAR against UVB-induced structural changes. Representative imaging of HaCaT cells untreated or treated with NAR (3.1 *μ*g/mL) for 24 hr, at 10 hr after UVB irradiation (55 mJ/cm^2^). (a) Confocal microscopy of HaCaT for nuclei (DAPI staining), F-actin (rhodamine-phalloidin staining), and CD95/Fas (anti-CD95/Fas rabbit pAb and secondary anti-rabbit antibody conjugated with rhodamine) (63x objective magnification). (b) SEM of non-UVB-exposed cells (1a, 1b), UVB-exposed cells (2a, 2b), and NAR-pretreated UVB-exposed cells (3a, 3b).

Changes in nuclei, F-actin cytoskeleton, and CD95/Fas cell surface receptor were studied by confocal laser scanning microscopy (Figure 4(a)). DAPI and rhodamine-phalloidin staining and anti-CD95/Fas immunostaining showed that UVB exposure causes, respectively, nuclei condensation and fragmentation with apoptotic body formation; impaired cell adherence and round cell shape; disruption of actin filament and weaker F-actin labelling; and CD95/Fas clustering with stronger labelling. Therefore, we confirmed that UVB exposure leads to characteristic apoptotic cell patterns. The NAR-pretreated cells exhibited a non-UVB-like cell shape with uniformity in cell nuclei (large and round), a highly polymerized F-actin filament, and a loss of anti-CD95/Fas clustering. In the presence of NAR, there was a marked absence of UVB-induced structural cell damages.

#### 3.4.2. SEM Cell Morphology

Cell surface morphology was observed by SEM imaging (Figures 4(b)). Non-UVB-exposed cultures showed a uniform cell layer including polygonal and flattened cells with numerous protrusions (Figures 4(b)1a and 4(b)1b), whereas UVB-exposed cultures exhibited various cell shapes—cell flattening and cell rounding with cytoplasmic retraction and membrane blebbing characteristic of apoptotic cells (white arrows) (Figures 4(b)2a and 4(b)2b). The NAR-pretreated UVB-exposed cells were mostly flattened and spread with protrusions (Figures 4(b)3a and 4(b)3b). Therefore, NAR protected cells against morphological features induced by UVB exposure.

#### 3.4.3. Cell Membrane Fluidity

**Figure 5:**
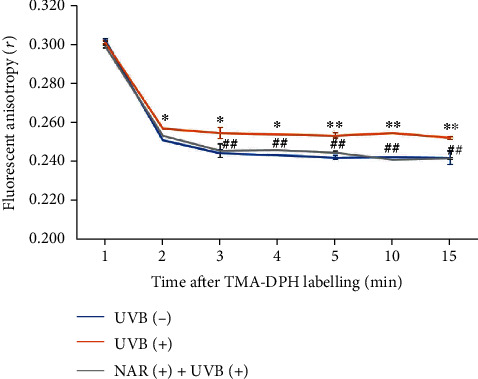
Protective effects of NAR versus UVB-influenced biophysical changes. Measurement of fluorescent anisotropy of TMA-DPH in HaCaT cells untreated or treated with NAR (3.1 *μ*g/mL) for 24 hr, at 2 min after UVB exposure (55 mJ/cm^2^). An experimentation was operated in triplicate (*n* = 3). Results are mean ± S.D. ^∗^*p* < 0.05 and ^∗∗^*p* < 0.01, compared between UVB (-) and UVB (+). ^##^*p* < 0.01, compared between UVB (+) and NAR (+) + UVB (+), using Student's unpaired *t*-test.

The fluidity of a membrane was appraised by means of the fluorescent anisotropy (*r*) of TMA-DPH, owing to the positive charge of its amino group, which is anchored at the surface of the cell membrane. The measurement of fluorescence polarization was used to determine the rotational diffusion of the probe (Figure 5). After 2 min of TMA-DPH labelling, fluorescence anisotropy values (*r*) of the UVB-exposed cells were significantly higher, in contrast to those of the non-UVB-exposed cells. This trend did not change during incubation time. Data showed that UV exposure induces a less fluid lipid bilayer and thereby alters the plasma membrane structure. The NAR pretreatment decreased the fluorescence anisotropy of TMA-DPH of UVB-exposed cells, with values close to those of non-UVB-exposed cells (at 4 min after labelling, *r* = 0.244 for non-UVB-exposed cells, *r* = 0.254 for UVB-exposed cells, and *r* = 0.246 for NAR-pretreated UVB-exposed cells). The NAR used on non-UVB-exposed cells did not affect to cell membrane fluidity (data not displayed). Thus, NAR prevented the loss of cell membrane fluidity after UVB exposure.

## 4. Discussion

**Figure 6:**
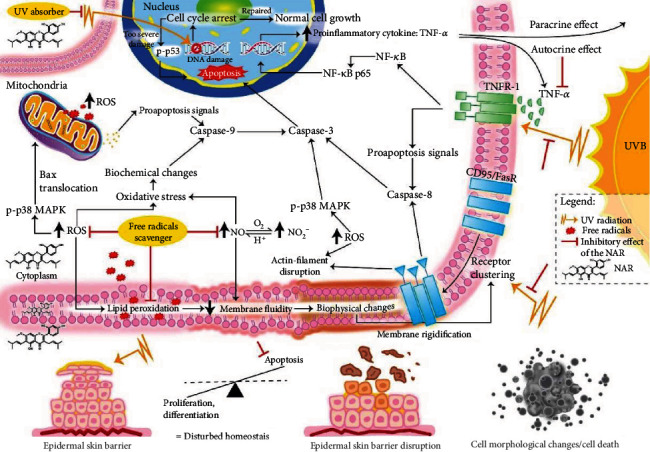
Schema displaying the mechanism of NAR against UVB-induced cell alterations. Exposure to UVB radiation induces massive apoptosis that contributes to skin photoaging and skin carcinogenesis. Apoptosis is an extremely regulated process that relates to a set of cellular events orchestrated mainly by the activation of caspases and leading to cell death. Signaling for UVB-induced apoptosis occurs through DNA damages and high levels of free radicals (ROS and NO), which initiate many cellular responses: (a) differential expression of p53 including halting of the cell cycle, repairing of DNA, and inducing cell death (apoptosis pattern); (b) p38 MAPK and NF-*κ*B pathway activation; (c) cell death membrane receptor activation (CD95/Fas) and proinflammatory cytokine secretion (TNF-*α*); and (d) damage to structural cell components (actin cytoskeleton, nucleus, and plasma membrane). NAR is a natural bioactive compound which is capable of both absorbing UV and scavenging free radicals, thereby showing an effective antioxidant capacity. It modulates intracellular signaling via direct interaction with free radicals, playing an antiapoptotic effect. It protects cells from the morphological and biochemical features of UVB-induced apoptosis, and therefore maintains skin homeostasis. We hypothesis that NAR is distributed in different cellular compartments as other antioxidant flavonoids, contributing to enhance its photoprotective effect.

The deleterious effects of excessive exposure to UVB bring about skin disorders: inflammatory skin, hyperplasia, premature aging of the skin, and skin cancer [4]. NAR is known as a natural flavonoid compound with a purity of around 99%, isolated from the *Artocarpus altilis* plant's heartwood by using diethyl ether. This active compound possesses several pharmacological properties and biological activities described in the literature: antioxidation [27–33], anti-inflammation [30, 32], tyrosinase and melanogenesis inhibition [27–29], and antimicrobial activity [34]. In this study, our results offer evidence that NAR is able to shield the detrimental effects of UVB irradiation. The biological basis for the efficacy of NAR was described and complemented our preceding findings (Figure 6).

Sunburn cells, an outstanding event caused by excessive exposure to UV, are due to the occurrence of apoptotic cells named keratinocytes, inducing death within the epidermis. Inappropriate apoptosis can contribute to skin malignancies and cancer; consequently, control of apoptosis is an important target for an effective photoprotection [7]. Here, we used an *in vitro* cell line HaCaT model to study the effect of NAR. Epidermal keratinocytes were irradiated with UVB at a single intensity of 55 mJ/cm^2^ to trigger apoptosis, following the procedure as shown by Luangpraditkun et al. in 2020 [33]. Sorting from low, moderate, and high amounts of UVB, the diverse features of cell death can be induced by running from apoptosis without inflammation, to apoptosis with a proinflammatory cytokine, and eventually up to necrosis with inflammation [35]. Our previous data showed that apoptosis induced with a physiological dose of 55 mJ/cm^2^ UVB was initiated by CPD lesions in DNA as well as ROS production, and mediated by caspase-3 and 8 activation [33]. In this work, we investigated other relevant markers of apoptosis to analyze the complete UVB response. Our analyses showed a significant overproduction of ROS and nitrite (nitrites are a metabolized form of NO reaction) following UVB expression and a significant reduction of these levels with the NAR pretreatment. We presented that the antioxidant effect of NAR is linked throughout its potentiality to scavenge ROS and NO. Being a free radical scavenger is an ordinary feature among different flavonoids and has been constituted by the hydroxyl groups to highly and reactively counteract to ROS and RNS [36, 37]. Flavonoids donate their hydrogen atoms for neutralizing free radicals. Flavonoid compounds thus display various antioxidant capabilities and have different impacts on free radical species. It is clear from the data of an *in vitro* study which demonstrated that the hydroxyl group figures of flavonoids are an eminent point for indicating their capabilities towards antioxidants and their effectiveness of free radical scavenging [38]. Furthermore, the overproduced ROS can interact with RNS such as NO to produce peroxynitrite anion (ONOO^−^) which is an extremely reactive radical. Studies have reported that ONOO^−^ activates apoptosis through p38 mitogen-activated protein kinases (MAPKs) and c-Jun N-terminal kinase (JNK) cascades. NO has been reported to degrade an antiapoptotic protein, namely, the myeloid cell leukemia 1 (Mcl-1) and activate dead cells via the apoptosis signal-regulating kinase- (ASK1-) JNK and BAX/BAK-activated caspase cascades [39]. Taken together, these data suggested that NAR confers direct antioxidant protection and thereby indirect antiapoptotic protection to cells.

Overproduction of intracellular ROS can overwhelm the antioxidant defenses and attack important cell components including membrane lipids, proteins, and DNA [20, 40]. Free radicals generated by UVB irradiation are implicated in oxidative cell membrane damages, such as membrane fluidity changes. Cellular membranes are highly dynamic structures. Thus, maintaining their structural integrity, their fluidity, and their rotational, translational, and transbilayer lipid movement is indispensable to preserve cell function [11, 41]. We used the TMA-DPH hydrophobic probe which incorporates instantaneously into the plasma membrane and becomes fluorescent. We measured its transbilayer movement characterizing the membrane fluidity. We showed that UV irradiation reduces the fluidity of the lipid membrane. This effect was due to the repression of the transbilayer phospholipid movement by free radicals [12]. Membrane phospholipids are easily oxidized by ROS and NO overproduction. Lipid peroxidation is a dominant hallmark to state the effect of UVB-induced skin disorders. We reported that the NAR pretreatment prevents membrane rigidification induced by ROS and nitrite. Our previous data supported that NAR reduces lipid peroxidation products (LPOs) *in vitro* [33]. In addition, it has been shown that a topical treatment with artocarpin on hairless mice before UVB exposure decreases LPOs [30]. Therefore, the degradation of membrane lipids generates pathological conditions in cells as a loss of membrane fluidity, an increase of membrane permeability, and eventually a breakdown of the plasma membrane causing cell death [15]. We confirmed that NAR alone or nonexposure to UV provides important antioxidant benefits, particularly in a lack of any indications of a significant change of membrane fluidity. Although the exact location of NAR in the lipid membrane is not precisely realized, we hypothesized that NAR intercalates the membrane lipid bilayer preferentially in the lipid rafts because most of the flavonoids, including NAR, are poorly water soluble.

A less fluid lipid bilayer event impedes transbilayer movement of phospholipids (flip-flop) and can induce clustering and activation of membrane receptors. CD95/Fas is an ubiquitously expressed cell surface receptor belonging to the TNF-R superfamily, involved in the apoptosis process. Our results validated that UVB irradiation activates the CD95/Fas receptor on HaCaT cells independently of the natural ligand CD95L, whereas the NAR pretreatment reduces its activation. Prevention of UVB-induced CD clustering by NAR was also associated with a preservation of cell morphology and a significant reduction of apoptosis markers. After UV exposure, we observed typical morphological features of apoptosis, such as a disruption of the actin cytoskeleton, nuclear condensation, membrane blebbing, and apoptotic body formation. In the presence of NAR, there was a marked reduction or absence of these hallmarks. It has been reported that ROS affect cytoskeleton proteins [42, 43]. Thus, the NAR pretreatment indirectly protected cytoskeletal components through its free radical scavenging potential. It has been demonstrated that the disrupted actin cytoskeleton can be conducted by activating CD95/Fas, and finally results in apoptosis [44]. It is well known that apoptosis affected by UVB is initiated by ROS as well as DNA damage and is mediated by a diverse array of signaling cascades, most of which are ultimately coupled to the activation of effector caspases. We found that NAR reduces the activation and thereby the activity of caspase-3, a crucial proteolytic enzyme of cell death signals, after UVB exposure. The function of many apoptosis-related signaling molecules, like p53, p38 MAPK, and NF-*κ*B p65 is regulated through posttranslational modifications, such as phosphorylation. Therefore, we studied the expression of these phosphorylated signal transducers and showed that they are stimulated when HaCaT cells are exposed to UVB and are so reduced among cells pretreated with NAR before exposing to UVB. Additionally, TNF-*α* is a critical regulator of both apoptosis and inflammation by mediating TNF receptor-associated intracellular signaling complexes [45]. Our data clarified that UVB radiation promotes TNF-*α* secretion by HaCaT cells, whereas the NAR pretreatment could decrease it. The antioxidant property by NAR mediated the inhibition of TNF-*α*-induced cell surface death receptor activation (TNF-R) through suppression of NF-*κ*B signaling. The paracrine effect of TNF-*α* cytokine from epidermal keratinocytes upregulated by UVB is associated with the loss of skin structural integrity by inducing dermal matrix metalloproteinases (MMPs) which have extracellular matrix-destructive potential [46]. Taken together, NAR could shield the detrimental effects of UV exposure by inducing the inhibition of apoptosis.

## 5. Conclusion

Exogenous antioxidants represent a safe and effective strategy to protect the human skin against UV-caused damage [47]. All the results of the present *in vitro* study highlighted the efficacy of NAR in epidermal photoprotection. NAR acts directly from scavenging free radicals generated by UV and indirectly by suppressing morphological and biochemical UV-induced cell damages. Its biological effects are mainly attributed to antioxidant and antiapoptotic properties. Thus, whereas natural products are popular in the field of biological sciences and pharmaceutical research [48, 49] and in the skincare market, NAR represents an effective natural ingredient in sunscreens.

## Data Availability

The data used to support the findings of this study have been deposited in the Hindawi repository [doi:10.1155/2020/1042451].
